# Assessment of Self-Management Care and Glycated Hemoglobin Levels Among Type 2 Diabetes Mellitus Patients: A Cross-Sectional Study From the Kingdom of Saudi Arabia

**DOI:** 10.7759/cureus.11925

**Published:** 2020-12-05

**Authors:** Bashair K Alshahri, Manar Bamashmoos, Mona I Alnaimi, Shaykhah Alsayil, Shymaa Basager, Mohammed T Al-Hariri, Christopher Amalraj Vallaba Doss

**Affiliations:** 1 Medicine, Imam Abdulrahman Bin Faisal University, Khobar, SAU; 2 Medicine, King Fahd University Hospital, Khobar, SAU; 3 Medicine, King Fahd University Hospital/Imam Abdulrahman Bin Faisal University, Khobar, SAU; 4 Physiology, Imam Abdulrahman Bin Faisal University, Khobar, SAU; 5 Statistics/Biostatistics, Imam Abdulrahman Bin Faisal University, Khobar, SAU

**Keywords:** self-management, type 2 diabetes mellitus

## Abstract

The prevalence of type 2 diabetes mellitus (T2DM) is increasing every year, along with its health and economic burden/impact. Achieving glycemic control remains challenging, and only 9-15% of diabetic patients manage to reach the optimal level. A few strategies have been found to improve diabetic control, including self-management care (SMC). This study aimed to explore the relationship between patient characteristics, SMC, and glycated hemoglobin (HbA1c) levels, as an indicator of optimal glycemic control. This was a cross-sectional study of 200 participants conducted at the King Fahd University Hospital (KFUH) in Saudi Arabia. A pre-structured questionnaire including sociodemographic data and aspects of diabetes self-management was distributed among patients at KFUH and the Family and Community Medicine Center (FAMCO) of Imam Abdulrahman Bin Faisal University, Dammam, Kingdom of Saudi Arabia. HbA1c data were extracted from patients' records. Unfortunately, the majority of the participants (65%) were found to have poor glycemic control. Glucose management was better in patients having T2DM for more than five years (mean: 4.01; p<0.05). In addition, an income of less than 5,000 Saudi Riyals (SR) was associated with lower physical activity (mean: 2.95; p<0.05). The level of blood sugar was uncontrolled among the majority of surveyed patients. Our study found variables associated with SMC and HbA1c levels, which might help to guide future initiatives aiming to enhance the care of patients with T2DM.

## Introduction

Globally, the prevalence of diabetes is increasing year by year. In 1980, there were 108 million diabetic patients worldwide, which quadrupled by 2014 [1]. In 2017, the number of diabetic patients aged 18-99 years was estimated to be around 425 million, and 49.7% of them were undiagnosed [2]. This number increased to 463 million cases by 2019, corresponding to 9.3% of adults in the total global population [3]. The prevalence continues to rise and is projected to reach 700 million cases by 2045 [3]. Despite the current efforts to curb it, diabetes accounted for five million deaths worldwide in 2017, representing 9.9% of all global mortality. Economically, diabetes cost the world USD 850 billion in 2017, and it is expected to increase to USD 958 billion by 2045 [2]. Previous studies have shown the extensive increase in the prevalence of type 2 diabetes mellitus (T2DM) and its related health implications, which is reflected in the enormous healthcare expenditure dedicated to managing T2DM [2,3]. Therefore, the provision of quality care for patients with T2DM is a priority for health systems across the world.

Successful provision of high-quality care to patients with T2DM is challenging. Only 9-15% of patients with T2DM achieve optimal glycemic control [4,5]. The role of the patient has changed from being a passive recipient to an active participant in the delivery of care [6]. Hence, diabetes management has become a comprehensive approach that involves lifestyle modifications, pharmacological therapy, and, sometimes, surgical interventions [7]. Therefore, patients' characteristics, self-care as well as medical treatments must be considered together with patients’ comorbidities, for controlling glycated hemoglobin (HbA1c) levels [8]. The present study explores the association of patients' characteristics with patients' self-care, and their HbA1c levels, in Saudi Arabia.

Epidemiology of diabetes mellitus in the Kingdom of Saudi Arabia

The Kingdom of Saudi Arabia is located in the Arabian Gulf region in the Middle East. The health system in Saudi Arabia is mainly operated by the Ministry of Health (MOH), with a contribution from the private sector, which amounts to 20-30% of the whole sector. Initiatives for patients with diabetes in the Kingdom operated by the department of disease control and prevention have different objectives; one of them is early detection of disease and preventing complications through good control of blood sugar levels. Despite these initiatives, the glycemic control among T2DM patients remains suboptimal [9]. According to the International Diabetes Federation (IDF), the prevalence of diabetes in the Middle East and North Africa region was 54.8% [6]. Saudi Arabia is ranked among the top 10 countries when it comes to the prevalence of T2DM, with prevalence rates exceeding the international rates. In 2013, the prevalence of diabetes was estimated to be 24% [10]. Financially, 6-16% of the global healthcare expenditure was devoted to diabetes, with the highest expenses seen in the Middle East and North Africa region [5]. In Saudi Arabia, the health expenditure for people diagnosed with diabetes compared to expenditure in the absence of diabetes is 10 times higher ($3,686 vs. $380) [11]. In addition, the actual economic burden is expected to be higher in the next future years. Hence, this study is an attempt to turn stakeholders' attention to cost-effective measures in managing this costly disease. Measures aimed at empowering diabetic patients by connecting them to the healthcare system, enabling them to have high-quality decisions, and equipping them with the responsibility for their own care should be implemented.

Patient characteristics and self-management care (SMC)

Patient demographic features are an important factor that is considered when discussing the care of diabetic patients. A vital aspect of chronic care and a cost-effective measure to maintain good diabetic control is self-management, which could be defined as the active participation of the patient in the attempt to achieve diabetic control [12]. Self-management involves several components, including adhering to medical management, emotional management such as handling frustration and sources of stress stemming from the nature of the chronic disease, and, finally, behavioral management, which includes complying with the dietary plan, blood glucose testing, pursuing routine physical activity, use of healthcare facilities, and attaining good overall rating related to diabetes self-care [12]. Poor self-management skills have been associated with poor glycemic control [13]. Few studies have explored SMC among diabetic patients in Saudi Arabia. Although most patients have reported good compliance with medication prescriptions [14], results have shown that patients had low compliance with other SMC practices like blood glucose testing, following a special diet, and exercise programs [14]. This reflects their poor understanding of the importance of these other measures in diabetes management. In fact, only a few patients reported having detailed information about SMC, while the majority were only given general instructions [14]. An experimental study in Saudi Arabia has shown that diabetes education has improved SMC practices and HbA1c and triglyceride levels [15]. In light of this, this study aims to explore the association between SMC and patient characteristics.

Patient characteristics and HbA1c

Patient demographic factors were reported with HbA1c of T2DM patients and were correlated with patient outcomes; for instance, race, diabetes duration, and age group also had an impact on the HbA1c levels [16]. Furthermore, the patient demographic factors related to T2DM considered by most of the studies were duration of diabetes, age, and body mass index (BMI) [17]. Research has found that investigating HbA1c predictors is beneficial in tailoring interventions to improve outcomes in diabetic patients [18]. Therefore, the present study aims to investigate diabetic patients' characteristics to inquire into possible future interventions by improving SMC, besides exploring the association of demographic factors with HbA1c levels in T2DM patients.

## Materials and methods

Design and sampling

This was a quantitative cross-sectional study conducted from October 2019 to April 2020 at the King Fahd University Hospital (KFUH) and the Family and Community Medicine Center (FAMCO) of Imam Abdulrahman Bin Faisal University, Eastern province, Kingdom of Saudi Arabia. This study included adult patients with T2DM, while patients with type 1 diabetes mellitus, other types of diabetes, and patients on their first visit were excluded. The sample was calculated for this study by using simple random sampling and was based upon diabetic patients' lists for those who had appointments or attended diabetic clinics in FAMCO and KFHU during the study period. The minimum required sample size at a 5% margin error, 50% assumed prevalence, and 95% confidence interval was 200, which was calculated by using Epi-info 7 Stat-Calc. The data were obtained via self-administered written and online voluntary questionnaires through telephone calls or a web-based survey in English and Arabic languages translated by experts to ensure their validity. The laboratory findings were obtained through the hospital's electronic health records via the patient medical record numbers (MRN).

Ethical considerations

The study was conducted after obtaining approval from the Institutional Review Board at Imam Abdulrahman University Hospital, Dammam, Saudi Arabia (IRB - UGS-2019-294) in October 2019. The questionnaire was distributed among patients who agreed to participate voluntarily in the survey. Ethical approvals for the use of the nine-item Shared Decision-Making Questionnaire (SDMQ-9) and the Diabetes Self-Management Questionnaire (DSMQ) were obtained from the original authors. Consent was obtained from all participants in this study.

Operationalization and instruments

SMC refers to the active participation of the patient to achieve diabetic control, and it involves several components including medical management, emotional management, and, lastly, behavioral management [12]. SMC is measured by DSMQ, which includes dietary control (Q5, Q13), glucose management (Q1, Q4, Q6), physical activity (Q8, Q11, Q15), healthcare use (Q3, Q7), and overall rating of diabetes self-care (Q16). For instance, patients were asked if they avoided physical activity, even though it would improve their diabetes; and their responses were rated on a 5-point scale (1 = applies to me very much; 2 = applies to me to a considerable degree; 3 = neutral; 4 = applies to me to some degree; 5 = does not apply to me) [19]. Glycemic control was measured by HbA1c levels. The data were obtained from electronic medical records. The criteria for good or bad control was based on the study by Ogbonna et al., and an HbA1c level of <7% was considered good control, and that of ≥7% was deemed poor control [20].

Data collection procedure

Recruitment of participants and data collection were conducted from January 2020 to March 2020. QuestionPro online survey software (QuestionPro, Austin, TX) was used to design the online questionnaire in English and Arabic languages. Data were obtained in this research from the survey and medical records. All patient characteristics were obtained from the questionnaire except for the laboratory data (lipid profile and HbA1c levels), which were obtained from medical records using patient MRN obtained from the surveys. The patient demographics considered in the questionnaire were age, sex, nationality, marital status, occupation, socioeconomic status, educational levels, duration of T2DM, comorbidities [hypertension (HTN) and dyslipidemia], BMI, and lipid profile including total cholesterol (TC), high-density lipoprotein (HDL), low-density lipoprotein (LDL) and triglycerides (TG). Moreover, SMC adherence data were obtained through DSMQ. The validation of the DSMQ showed Cronbach's alpha reliability scores of 0.651 for 11 previously mentioned questions out of 16 with factor loading higher than 0.6.

Data analysis

Before sending data to the biostatistician, we audited, coded, organized, and anonymized the data. Descriptive statistics were used to summarize patient demographic and clinical characteristics. Patient BMI was calculated by dividing the patient weight in kilograms by the height of the patient in square meters. Patients were assigned different BMI status based on the WHO classification of BMI (i.e., BMI of ≤18.5: underweight; BMI of 18.5-24.9: normal; BMI of 25.0-29.9: overweight; and BMI of ≥30.0: obese). Lipid profile was categorized as follows: TC (controlled: <200 mg/dl, uncontrolled: ≥200 mg/dl); HDL (uncontrolled: <40 mg/dl, controlled: ≥40 mg/dl); TG (normal: <150 mg/dl, borderline: 150-199 mg/dl, high: 200-499 mg/dl); LDL (controlled: <100 mg/dl, uncontrolled: ≥100 mg/dl) [21]. We used SPSS Statistics version 21 (IBM, Armonk, NY) for statistical analysis. A p-value of <0.05 was considered statistically significant. Analysis of variance (ANOVA) and t-tests were used to identify demographic factors associated with SMC. Logistic regression was used to test the impact of patients’ characteristics on HbA1c levels.

## Results

A sample of 200 diabetic patients with T2DM was enrolled in the study, while 18 patients were excluded because they had other types of diabetes. Hence, the analysis was performed on the 200 T2DM patients (92%) who completed the questionnaires. A total of 400 surveys were distributed, which had a 50% response rate.

Participant characteristics

Sociodemographic characteristics of the diabetic patients including age, sex, nationality, marital status, occupation, socioeconomic status, educational levels, duration of T2DM, comorbidities (HTN and dyslipidemia), BMI, and lipid profile including TC, HDL, LDL, and TG are shown in Table 1. Participants were classified into four age groups, where most of the participants (39%) were above or equal to 60 years old. Of the study sample, 50% were female. The majority of participants were Saudi (85%) and married (83%). Regarding socioeconomic status and educational level, most of the sample (69.5%) had an average monthly income of 5,000 Saudi Riyals (SR) or above, and most of them were literate (86.5%). About 39.5% were employed. The majority of the subjects (75%) were being followed up at KFUH, while only 11.5% were being followed up at FAMCO; 75.5% of the subjects had comorbidities, which included HTN, dyslipidemia, or both. Approximately more than half of the sample (80%) were obese or overweight, with only 13.5% of patients having a normal BMI. Furthermore, 76% were having T2DM for more than five years. As for laboratory results, 65% of the sample had poor control of HbA1c (≥7%). Regarding the lipid profile, surprisingly, most of the participants had good control of TC, HDL, and TG, accounting for 67%, 86%, and 37.5% respectively, while LDL was poorly controlled among 46% of the sample.

**Table 1:** Participant characteristics and labs T2DM: type 2 diabetes mellitus; BMI: body mass index; HbA1c: glycated hemoglobin; TC: total cholesterol; TG: triglycerides; LDL: low-density lipoprotein; KFUH: King Fahd University Hospital; FAMCO: Family and Community Medicine Center; SR: Saudi Riyal; HTN: hypertension

Characteristics		N	%
Gender	Male	100	50.0
	Female	100	50.0
Age	<40 years	17	8.5
	40-49 years	36	18.0
	50-59 years	69	34.5
	≥60 years	78	39.0
Occupation	Employed	79	39.5
	Unemployed	117	58.5
	Student	4	2.0
Duration of T2DM	<5 years	48	24.0
	≥5 years	152	76.0
Healthcare center	KFUH	150	75.0
	FAMCO	23	11.5
	Missing	27	13.5
Nationality	Saudi	171	85.5
	Non-Saudi	29	14.5
Marital status	Married	166	83.0
	Single	15	7.5
	Divorced	9	4.5
	Widowed	10	5.0
Socioeconomic status (income)	Less than 5,000 SR/month	61	30.5
	5,000-10,000 SR/month	64	32.0
	More than 10,000 SR/month	75	37.5
Educational levels	No degree	28	14.0
	High school	87	43.5
	Diploma	22	11.0
	Bachelor's degree	48	24.0
	Postgraduate	15	7.5
Comorbidity	Nothing	48	24.0
	HTN	50	25.0
	Dyslipidemia	53	26.5
	HTN and dyslipidemia	49	24.5
BMI	Normal	27	13.5
	Overweight	71	35.5
	Obese	89	44.5
	Missing	13	6.5
HbA1c	Good (<7%)	43	21.5
	Poor (≥7%)	130	65.0
	Missing	27	13.5
TC	Controlled (<200 mg/dL)	134	67.0
	Uncontrolled (≥200 mg/dL)	40	20.0
	Missing	26	13.0
TG	Normal (<150 mg/dL)	75	37.5
	Borderline (150-199 mg/dL)	59	29.5
	High (200-499 mg/dL)	39	19.5
	Missing	27	13.5
LDL	Controlled (<100 mg/dl)	80	40.0
	Uncontrolled (≥100 mg/dl)	93	46.5
	Missing	27	13.5

The association between participant characteristics and adherence to self-management care

Independent t-test and ANOVA test were used to test the association between patient characteristics and adherence to SMC (Table 2). Primarily, socioeconomic levels of less than 5,000 SR monthly and educational level of no degree were significantly associated with lower physical activity (mean: 2.95, p<0.05), (mean: 2.59, p<0.001) respectively. Secondly, the comorbidity of HTN was significantly associated with higher physical activity (mean: 3.62, p<0.01). Thirdly, there was a statistically significant association between normal BMI and higher physical activity (mean: 3.59, p<0.05). Also, the lower use of the healthcare system was associated with the age group of younger than 40-year-olds, employed group, and normal BMI group (mean: 4.29, p<0.05), (mean: 4.50, p<0.05), (mean: 4.33, p<0.05) respectively. Furthermore, there was a significant association indicating that the usage of the healthcare system was more prevalent among Saudi nationals (mean: 4.72, p<0.001). Higher dietary control was significantly associated with patients having T2DM for more than five years (mean: 3.85, p<0.05). Moreover, higher dietary control was significantly associated with unemployed patients (mean: 3.77, p<0.05). In addition, higher self-glucose management was significantly associated with unemployed patients (mean: 3.95, p<0.05) and with patients having T2DM for more than five years (mean: 4.01, p<0.05). Finally, the male gender was significantly associated with higher physical activity (mean: 3.61, p<0.001).

**Table 2:** Association between participant characteristics and adherence to self-management care The asterisk represents statistically significant differences: *p<0.05, **p<0.01, ***p<0.001; ^independent t-test; ^#^ANOVA test T2DM: type 2 diabetes mellitus; SR: Saudi Riyal; BMI: body mass index; TC: total cholesterol; HDL: high-density lipoprotein; LDL: low-density lipoprotein; KFUH: King Fahd University Hospital; FAMCO: Family and Community Medicine Center; HTN: hypertension; SD: standard deviation; ANOVA: analysis of variance

Characteristics		Dietary control		Glucose management		Physical activity		Healthcare use		Overall rating of diabetes self-care	
		Mean	SD	Mean	SD	Mean	SD	Mean	SD	Mean	SD
Gender	Male	3.78	1.09	3.94	1.05	3.6**^	1.12^	4.64	0.63	4.00	1.32
	Female	3.67	1.10	3.89	0.97	2.99^	1.19^	4.64	0.69	3.85	1.31
Age	<40 years	3.26	1.30	3.72	1.31	3.66	1.37	4.29*^#^	0.90^#^	4.00	1.41
	40-49 years	3.44	1.14	3.73	1.01	3.23	1.07	4.45^#^	0.85^#^	3.75	1.33
	50-59 years	3.78	1.07	4.00	0.97	3.35	1.13	4.71^#^	0.55^#^	3.86	1.37
	≥60 years	3.90	1.02	3.96	0.96	3.21	1.26	4.74^#^	0.56^#^	4.03	1.25
Occupation	Employed	3.72*^#^	1.00^#^	3.92*^#^	1.06^#^	3.44	1.10	4.50*^#^	0.74^#^	3.89	1.33
	Unemployed	3.77^#^	1.13^#^	3.95^#^	0.92^#^	3.18	1.25	4.73^#^	0.60^#^	3.92	1.32
	Student	2.37^#^	1.37^#^	2.58^#^	1.52^#^	4.08	1.06	4.87^#^	0.25^#^	4.50	1.00
Duration of T2DM	<5 years	3.31*^	1.24^	3.59*^	1.13^	3.34	1.29	4.47	0.83	4.00	1.35
	≥5 years	3.85^	1.02^	4.01^	0.95^	3.28	1.17	4.69	0.59	3.90	1.31
Healthcare center	KFUH	3.73	1.10	3.95	1.00	3.28	1.18	4.70	0.60	3.92	1.28
	FAMCO	3.63	0.84	3.84	1.00	3.50	1.14	4.52	0.69	4.13	1.28
Nationality	Saudi	3.76	1.08	3.91	1.05	3.30	1.23	4.72***^	0.57^	3.96	1.29
	Non-Saudi	3.48	1.19	3.94	0.75	3.28	1.00	4.15^	0.93^	3.68	1.46
Marital status	Married	3.71	0.08	3.95	0.07	3.30	0.09	4.66	0.04	3.90	0.10
	Single	3.86	0.30	3.71	0.32	3.53	0.29	4.63	0.16	4.33	0.25
	Divorced	3.94	0.29	3.59	0.45	3.22	0.39	4.16	0.34	4.11	0.45
	Widowed	3.50	0.42	3.86	0.31	3.06	0.46	4.75	0.20	3.50	0.47
Socioeconomic status (income: SR/month)	<5,000	3.58	1.20	3.83	1.02	2.95*	1.30	4.51	0.80	3.85	1.42
	5,000-10,000	3.75	1.05	4.06	0.84	3.41	1.06	4.69	0.60	3.87	1.16
	>10,000	3.82		3.85	1.12	3.49	1.16	4.70	0.58	4.02	1.36
Educational levels	No degree	3.37	1.20	3.84	0.89	2.59***	1.13	4.62	0.71	3.85	1.29
	High school	3.77	1.07	3.79	1.13	3.15	1.23	4.67	0.66	3.80	1.31
	Diploma	3.63	0.90	3.95	0.79	3.57	1.04	4.81	0.36	3.95	1.21
	Bachelor's degree	3.78	1.23	4.01	0.98	3.62	1.06	4.61	0.69	3.93	1.47
	Postgraduate	4.10	0.80	4.37	0.74	4.04	.958	4.33	0.79	4.66	0.81
Comorbidity	Nothing	3.63	1.05	3.86	0.97	3.30	1.08	4.60	0.72	3.91	1.25
	HTN	3.79	1.20	4.12	0.95	3.62**	1.13	4.65	0.59	4.02	1.39
	Dyslipidemia	3.73	1.07	3.70	1.10	3.44	1.15	4.66	0.59	3.81	1.33
	HTN and dyslipidemia	3.71	1.09	3.97	0.97	2.78	1.28	4.64	0.77	3.93	1.32
BMI	Normal	3.98	0.23	3.83	0.22	3.59*^#^	0.19^#^	4.33*^#^	0.15^#^	4.00	0.26
	Overweight	3.74	0.12	3.95	0.11	3.48^#^	0.14^#^	4.75^#^	0.06^#^	3.85	0.15
	Obese	3.54	0.12	3.90	0.10	3.03^#^	0.12^#^	4.65^#^	0.07^#^	3.95	0.13
TC	Controlled (<200 mg/dl)	3.72	0.09	3.99	0.08	3.34	0.10	4.66	0.05	3.92	0.11
	Uncontrolled (≥200 mg/dl)	3.70	0.16	3.67	0.18	3.15	0.18	4.67	0.10	3.82	0.20
HDL	Controlled (≥40 mg/dl)	3.72	1.07	3.92	1.00	3.31	1.17	4.68**	0.61	3.90	1.32
	Uncontrolled (<40 mg/dl)	3.50	-	3.66	-	2.33	-	3.00	-	3.00	-
LDL	Normal (<150 mg/dl)	3.57	1.12	3.98	0.95	3.23	1.25	4.69	0.64	3.86	1.22
	Borderline (150-199 mg/dl)	3.74	1.07	3.81	1.06	3.46	1.01	4.59	0.71	3.77	1.46
	High (200-499 mg/dl)	3.94	0.94	3.94	1.02	3.17	1.25	4.73	0.44	4.12	1.26

The association between participant characteristics and good HbA1c levels

Logistic regression model was used to predict the level of HbA1c based on patients’ characteristics (Table 3). Female gender and uncontrolled TC or uncontrolled LDL were significant predictors of poor HbA1c (B = 0.153; 95% CI = 1.1-1.4), (B = .202; 95% CI = 1.039-1.440) (B = .176; 95% CI = 1.024-1.388) respectively. While higher educational levels [bachelors degree (B = -.300; 95% CI = .574-.957) and postgraduate degree (B = -.582; 95% CI = .357-.875)] were significant predictors of better HbA1c.

**Table 3:** Association between participant characteristics and good HbA1c levels The asterisk (*) represents statistically significant association (p-value of <0.05 between the SDM among the variables) HbA1c: glycated hemoglobin; BMI: body mass index; TC: total cholesterol; HDL: high-density lipoprotein; TG: triglycerides; LDL: low-density lipoprotein; SR: Saudi Riyal; HTN: hypertension

		HbA1c	
		Unadjusted mode	
Patient characteristics	Classifications	B (95% CI)	P-value
Gender	Male	Reference	0.04*
	Female	0.153 (1.1-1.4)	
Age	<40 years	Reference	-
	40-49 years	-.043 (.725-1.265)	.760
	50-59 years	-.080 (.712-1.197)	.545
	≥60 years	-.115 (.689-1.152)	.380
Occupation	Employed	Reference	-
	Not employed	.069 (.925-1.242)	.356
	Student	.096 (.655-1.851)	.717
Duration of T2DM	<5 years	Reference	-
	≥5 years	.187 (.995-1.460)	.057*
Nationality	Saudi	Reference	-
	Non-Saudi	-.078 (.755-1.134)	.456
Marital status	Married	Reference	-
	Single	.080 (.822-1.426)	.571
	Divorced	.217 (.925-1.668)	.150
	Widowed	.025 (.759-1.384)	.871
Socioeconomic status (income)	Less than 5,000 SR/month	Reference	-
	5,000-10,000 SR/month	.035 (.877-1.224)	.679
	More than 10,000 SR/month	-.147 (.719-1.037)	.117
Educational levels	No degree	Reference	-
	High school	-.005 (.814-1.216)	.959
	Diploma	-.148 (.641-1.161)	.329
	Bachelor's degree	-.300 (.574-.957)	.021*
	Postgraduate	-.582 (.357-.875)	.011*
Comorbidity	HTN	Reference	-
	Dyslipidemia	-.037 (.786-1.183)	.725
	HTN and dyslipidemia	.056 (.871-1.283)	.572
	Nothing	.020 (.819-1.222)	.998
	Other	-.416 (.159-2.729)	.566
BMI	Normal	Reference	-
	Overweight	-.121 (.708-1.108)	.288
	Obese	-.041 (.777-1.186)	.704
TC	Controlled (<200 mg/dl)	Reference	-
	Uncontrolled (≥200 mg/dl)	.202 (1.039-1.440)	.015*
HDL	Controlled (≥40 mg/dl)	Reference	-
	Uncontrolled (<40 mg/dl)	-.241 (.240-2.577)	.691
TG	Normal (<150 mg/dL)	Reference	-
	Borderline (150-199 mg/dL)	.007 (.857-1.183)	.930
	High (200-499 mg/dL)	-.152 (.699-1.055)	.148
LDL	Controlled (<100 mg/dl)	Reference	-
	Uncontrolled (≥100 mg/dl)	.176 (1.024-1.388)	.024*

## Discussion

The main goal of diabetes management is to ensure optimal glycemic control. The present work studied the association between the level of glycemic control and its related factors among T2DM patients. The results of this study showed that approximately two-thirds of patients with T2DM had poor glycemic control, which corresponds with MOH's recent publication, which found that despite all current initiatives, glycemic control among T2DM patients is still suboptimal [9]. Also, another study conducted at Jazan showed that 74.3% of the participants had poor glycemic control (HbA1c of <7%), which was similar to another study among the Saudi population in a different city [22]. This significant prevalence of poor glycemic control in the country reinforces the need to explore factors and initiatives that would have a considerable effect on glycemic control in T2DM patients.

Regarding factors associated with adherence to a diabetic diet, the results did not reveal an association between educational levels and dietary adherence. However, previous studies found higher educational levels to be associated with better compliance with a dietary regimen; for instance, patients with no degree had lower physical activity and vice versa [23]. This result could be attributed to the fact that a high educational level does not necessarily translate into good knowledge about the prevention and control of T2DM. This could also be due to the eating culture in Saudi Arabia, which makes it hard to adhere to a diabetic diet even among highly educated people. Patients who were diagnosed for three to five years were most likely to follow a diabetic diet [24]. Likewise, this study showed a significant association between patients having T2DM for more than five years and adherence to diet and better self-glucose management. Regarding physical activity, males were found to be more physically active than females [25]. In addition, Saudi females were disproportionately less active than males, starting from their school years, which could be attributed to cultural barriers, lack of social support, and absence of exercise programs in female schools [26]. Moreover, consistent with this study's findings, one study showed that the lower the socioeconomic level, the higher the rate of inactivity [25]. Also, a study conducted at a university hospital in Riyadh showed that the major barriers to exercise were lack of resources, especially among low-income people [26]. Somewhat surprisingly, individuals who were comorbid with HTN were physically more active. In contrast with the present paper, healthcare system use was found more common among younger patients who were less than 45 years old in one study [27], while this study revealed low healthcare system use among patients aged less than 40 years.

In this study, HbA1c was significantly higher among patients with uncontrolled LDL and TC. This finding is consistent with that of previously published studies. However, in this study, HDL and TG were not significant predictors of HbA1c levels [28]. Regarding gender, being a female was a significant predictor of higher HbA1c levels, which can be explained by the high prevalence of obesity, lack of activity, and unhealthy lifestyle among Saudi females [29]. In contrast, MA et al. reported in their study that male patients had significantly elevated HbA1c levels [30], while Kakade et al.'s study showed no significant difference between genders [28]. Similar to Badedi et al.'s study, a higher educational level had a protective effect [22]. Both bachelor's and postgraduate degree holders had lower HbA1c levels. Likewise, patients with lower educational levels had poorly controlled diabetes [28].

Limitations

This study has some limitations. Primarily, this was a cross-sectional study, which did not provide a causal relationship. Secondly, findings from a sample size of 200 T2DM patients collected from two institutions in the Eastern Province may not be generalizable to a wider populace. Thirdly, the fact that our sample consisted of people visiting clinics may have led to the underrepresentation of certain underprivileged groups who do not have access to healthcare facilities. Therefore, future research may adopt a different study design, which can provide a causal relationship. In addition, involving a broad range of institutions, including both private and governmental, may help in attaining data that could be generalized to the whole of Saudi Arabia. Moreover, future initiatives can utilize information technology for optimizing the self-care of patients with T2DM, by setting reminders and alarming systems for patients and/or treating patients remotely.

## Conclusions

Controlling blood glucose is crucial in the management of T2DM to avoid complications. Unfortunately, this study showed that the majority of T2DM patients had poor glycemic control. Therefore, patients need healthcare professionals to educate and encourage them to adopt SMC techniques due to their valuable impact on the management of diabetes. We believe this study will persuade Saudi healthcare authorities to formulate new strategies to educate T2DM patients about proper SMC. We also recommend that interventions be made as early as possible since newly diagnosed people are more eager to learn. Finally, we hope this study will encourage governments and stakeholders to introduce sports programs in female schools.

Based on the limitations of this study, there is a need for further studies to comprehensively determine the association of patients’ characteristics and SMC with glycemic control, taking into consideration the sample size and study design.
